# Weight, height and eruption times of permanent teeth of children aged 4–15 years in Kampala, Uganda

**DOI:** 10.1186/1472-6831-13-15

**Published:** 2013-03-16

**Authors:** Annet Kutesa, Eriab Moses Nkamba, Louis Muwazi, William Buwembo, Charles Mugisha Rwenyonyi

**Affiliations:** 1 Department of Dentistry College of Health sciences, Makerere University, P.O. Box 7072, Kampala, Uganda; 2 Department of Anatomy, College of Health sciences, Makerere University, Kampala, Uganda

**Keywords:** Height, Permanent teeth, Tooth eruption age, Uganda, Weight

## Abstract

**Background:**

Tooth eruption is a continuous biological process by which developing teeth emerge through the jaws and the overlying mucosa to enter into the oral cavity. Tooth eruption time and sequence are important factors in dental treatment planning, particularly in orthodontics, but also in forensic dentistry to estimate age of a child. Tooth eruption time is influenced by many factors. In this study we set out to determine the timing of eruption of permanent teeth and assess its association with the height and weight of school children aged 4–15 years in Kampala, Uganda.

**Methods:**

This was a cross sectional study comprising of 1041 healthy Ugandan children: boys/girls (520/521) who were consecutively selected from two primary schools in Kampala. The children were clinically assessed for tooth emergency through the oral mucosa as well as measuring their weight and height. The mean and standard deviation of tooth eruption time was estimated for boys and girls. Bivariate analysis was used to assess any significant association between tooth eruption time and demographic variables. Pearson and partial correlation analyses were used to assess any significant association between the tooth eruption time and anthropometric measurements of the children.

**Results:**

Generally, the mean eruption times for girls were lower compared to boys except for three teeth (#25, #32 and #42) which erupted earlier in boys. The average difference in mean eruption times of all teeth between boys and girls was found to be 0.8 (range, 0–1.5) years. In partial correlation analysis, mean tooth eruption times were positively, but not significantly associated with height while controlling for weight except for the mandibular left central incisor (#31). On the other hand, in partial correlation analysis, mean tooth eruption times were positively associated with weight while controlling for height except for tooth #11, #16, #26 and #41. The weight of the child was significantly correlated with mean eruption times in 50% of the teeth.

**Conclusion:**

In the present study, the mean tooth eruption times for girls were lower compared to boys except for three teeth (#25, #32 and #42). The height of the child did not show any significant influence on the tooth eruption times while the influence of weight on tooth eruption times was non-conclusive.

## Background

Tooth eruption time and sequence are important factors in dental treatment planning especially in orthodontics, but also in forensic dentistry to estimate age of a child. Tooth eruption is a continuous biological process by which developing teeth emerge through the jaws and the overlying mucosa to enter into the oral cavity and contact the teeth of the opposing arch [1]. Several studies [2,3] have shown that eruption of the permanent teeth is orderly, sequential and age-specific event. Generally, permanent teeth have been found to erupt between the ages of 5 to 13 years, except the third molars which do so between 17 and 21 years [2,3]. Furthermore, tooth eruption time as well as the sequence of tooth eruption have been reported to vary with races [2,4-6].

It has also been reported that some other variables like genetics, hormonal factors, geographical location, ethnicity, sex, economic status, nutrition and growth exert their influences on tooth eruption time [7,8]. A few studies have indicated a relationship between the eruption times with the weight and height of the children. Children who are below average weight and height have been shown to have a later eruption times than those who are within the standard range [4,9]. Khan [10] reported that tall children exhibited delayed tooth eruption irrespective of their weight while heavy and short children had early eruption. Agarwal [2] found that for the same age group, boys with more sexual maturity had enhanced dental eruption, in support of the earlier findings [11,12].

Most of the literature on dental development is essentially from outside Africa, which may not be applicable to the African population due to racial and environmental differences. The only study [13] on tooth eruption among Ugandan children was done more than 40 years ago, over which environmental and socioeconomic changes have taken place. The purpose of this study was to determine the timing of eruption of permanent teeth and assess its association with height and weight of children aged 4 to15 years in Kampala, Uganda.

## Methods

### Study setting

This was a cross sectional study in randomly selected schools within Kampala. Kampala is the metropolitan city of Uganda and comprises of five administrative municipalities namely; Kawempe, Central, Lubaga, Makindye and Nakawa. The study took place in two randomly selected municipalities: Lubaga and Makindye. The two municipalities have an estimated day population of about 900,000 people. The population is multi-ethnic with people from all districts of Uganda. A list of schools with kindergarten and primary sections within the two selected municipalities was obtained from Kampala City Council Authority Education office. One school was randomly selected from each of the municipalities: Wabigalo Day and Boarding School in Makindye Municipality and Happy Years Primary School, Kabuusu in Lubaga Municipality.

### Subject selection

The children were recruited consecutively from the two schools. The selection criteria were that a child had to be a healthy Ugandan of African decent, aged 4 to15 years and consented to participate in the study. The age and nationality of the child were ascertained from school records where 3 children were excluded for being nonUgandans. In order to be considered healthy, a child had no history of systemic or chronic disease(s), thus 4 children were excluded: 1 for having epilepsy; 1, oral cleft and 2 sickle cell disease leaving a total of 1041 children for the study.

### Ethical considerations

Ethical approval was obtained from Makerere University School of Medicine Institutional Review Board. Permission to carry out the study was obtained from Kampala City Council Authority Education office and the respective heads of schools. Written consent was obtained from the parents/guardians on behalf of the children and assent was also obtained from the study children in accordance with Helsinki Declaration [14].

### Calibration of examiners

Prior to the field survey, four dentists (research assistants) were trained by a human anatomist (WB) in data collection on children (n = 25) of St. Martin Primary School, Kampala. They were trained in clinical assessment of an erupted and not erupted tooth. They also trained in the measurement of height and weight of the children. The mean inter-examiner consistence in recording tooth emergence was 100%; weight, 96% (range, 94%–98%) and height, 95% (range, 93%–98%).

### Data collection

The data were collected from the study children by 4 research assistants who had previously been calibrated in the data collection. The demographic information included sex and date of birth. The date of birth was verified from the school records and was used to compute the age of the child. Dental examination of the children was conducted in broad day light with the help of a plane mouth mirror and pair of tweezers. The child was lying supine on a school wooden bench with the head resting on the examiner’s lap who was seated at 12 o’clock position and inclined forward to fully access the child’s oral cavity. The teeth were cleaned of food debris with cotton wool for proper visibility. Each permanent tooth was recorded using the two digit system of the *Fedération Dentaire Internationale* notation [15]. The emergence stages of the teeth into the oral cavity are classified into four stages [5]. The stages are defined as 0 = the tooth not visible in the oral cavity; 1 = at least one cusp visible in the oral cavity; 2 = the entire occlusal surface/mesio-distal width of the tooth visible; and 3 = the tooth in occlusion or at the occlusal level if the antagonistic tooth was not fully erupted or missing. For purposes of the present study, we recorded a tooth as having erupted when any part of the crown was visible through the oral mucosa (i.e. stages 1, 2 and 3) defined as category 1 and when not erupted = 0. Recording of extracted teeth due to caries was based on World Health Organisation guidelines [16]. Wisdom teeth were excluded. The study was school based and could not assess any congenitally missing teeth due to lack of appropriate radiological facilities like x-rays.

The child’s physical body development was assessed through anthropometric measurements. The weight was determined by weighing a child in kilograms using a digital weighing scale (Avery, United Kingdom) after removal of shoes and any overcoat. The values were corrected to the nearest one decimal point. The height of the child was measured when the child was standing while the back and knees were straight, and feet drawn together. The line of vision was in the horizontal plane. Then the height was measured from the heel to the uppermost part of the head using a wall mounted tape measure (Avery, United Kingdom) in metres and corrected to the nearest one decimal point.

### Reliability test

There was no attempt to evaluate the error of the method of recording tooth eruption because the criteria for tooth emergence are so clear [17]. Blind duplicate measurement of the weight and height of about 10% (n = 102) children was done to assess reproducibility of the 4 examiners at an interval of 7 days. This was based on systematic random selection of every 10^th^ child from the study sample. The intra-class correlation coefficients were calculated to check for consistency [18] in the anthropometric measurement of 102 children. The agreement was almost perfect with correlation coefficients between 0.85 and 0.96 for the weight and height of the children with no evidence of systematic error observed (>0.05; paired t test).

### Data analysis

The data were analyzed using the Statistical Package for Social Science Inc. (SPSS, version 17 for windows, Chicago, Illinois, USA). The intra-class correlation analysis was used to check for consistency in the anthropometric measurement of the children. Student’s t test for paired observations was used to check for systematic errors in anthropometric measurements. The values of extracted teeth were converted to category 1, i.e. tooth having erupted. The eruption times were computed based on the technique previously described [15,19]. The mean and standard deviation of tooth eruption times were estimated for boys and girls. The standard error of the mean was calculated using the delta method [20]. Bivariate analysis was used to assess any association between tooth eruption times and the quantitative variables. Pearson and partial correlation analyses were used to determine any association between the mean tooth eruption times and anthropometric measurements. Body mass index (BMI) of each child was determined as the ratio of weight (in kg) to the square of height (in m). The likelihood ratio tests were used to assess any significant differences in mean eruption times between contra-lateral teeth of right and left quadrants as well as antagonistic teeth in mandible and maxilla.

Student’s t test for independent samples was used to test whether the correlation coefficient was significantly different from zero and to test differences between means of quantitative variables. The level of significance was set at 5%.

## Results

A total of 1041 children aged 4–15 years were consecutively recruited for the study. The overall mean age of the children was 8.8 ± 3.0 years (boys, 9.0 ± 3.1; girls, 8.6 ± 3.0, Table 1). The distribution of boys and girls based on chronological age was not significantly different (p >0.05).

**Table 1:** The frequency distribution of age and sex of the study children (n = 1041)

**Age (years)**	**Boys**	**Girls**	**All**
4	42	44	86
5	55	53	108
6	52	72	124
7	34	44	78
8	32	44	76
9	49	46	95
10	53	52	105
11	71	46	117
12	57	67	124
13	47	38	85
14	22	10	32
15	6	5	11
All	520	521	1041

Generally, the mean eruption times for girls were lower compared to boys except for three teeth (#25, #32 and #42) which were found to erupt earlier in boys (Table 2). The average difference in mean eruption times of all teeth between boys and girls was 0.8 (range, 0–1.5) years. The standard error of the mean ranged from 0.1 to 0.9 years for boys and 0.1 to 0.6 for girls (Table 2).

**Table 2:** The frequency distribution of number of cases, mean, standard deviation (SD) and standard error of the mean (SEM) of tooth eruption age (years) of boys and girls according to tooth types

**Tooth number**	**Boys (n = 520)**				**Girls (n = 521)**				**All (n = 1041)**			
	**No. of cases**	**Mean**	**SD**	**SEM**	**No. of cases**	**Mean**	**SD**	**SEM**	**No. of cases**	**Mean**	**SD**	**SEM**
11	69	6.3	0.8	0.3	48	6.1	2.1	0.9	117	6.2	1.5	0.5
12	63	8.5	2.1	0.5	57	7.1	0.9	0.3	120	7.4	1.2	0.4
13	143	10.9	1.2	0.2	72	9.4	1.2	0.3	215	10.4	1.4	0.2
14	57	9.6	1.1	0.3	46	9.1	1.4	0.5	103	9.4	1.2	0.3
15	42	10.0	1.0	0.4	26	9.6	1.3	0.5	68	9.8	1.2	0.4
16	30	6.0	1.4	0.4	25	5.0	0.2	0.1	55	5.4	0.9	0.4
17	49	10.8	1.0	0.5	32	10.5	1.3	0.5	81	10.6	1.2	0.3
21	67	6.3	1.5	0.3	49	6.2	0.4	0.2	116	6.2	0.8	0.3
22	65	8.4	1.4	0.5	51	7.2	1.0	0.3	116	7.8	1.3	0.3
23	139	10.6	1.2	0.2	66	9.1	1.0	0.2	205	10.0	1.3	0.2
24	59	9.5	1.3	0.4	50	9.4	1.3	0.4	109	9.4	1.3	0.3
25	49	9.3	1.8	0.3	33	10.5	1.0	0.5	82	9.8	1.5	0.5
26	30	6.7	2.0	0.4	26	5.5	1.0	0.5	56	6.3	1.8	0.5
27	50	11.2	3.2	0.6	32	11.0	1.2	0.4	82	11.1	2.0	0.5
31	44	6.0	0.9	0.2	21	5.5	2.7	0.9	61	5.7	2.1	0.5
32	62	5.7	0.5	0.2	43	6.6	0.8	0.3	105	6.1	6.1	0.2
33	72	10.0	1.7	0.4	58	10.0	2.5	0.9	130	10.0	2.0	0.4
34	55	10.0	1.7	0.5	45	9.1	1.0	0.3	100	9.4	1.3	0.3
35	35	10.5	1.0	0.1	23	10.3	1.1	0.4	58	10.4	1.1	0.3
36	44	5.8	1.2	0.3	34	5.3	0.5	0.2	78	5.5	0.8	0.3
37	85	11.5	1.7	0.4	63	6.1	2.1	0.9	148	6.2	1.5	0.5
41	43	7.0	0.2	0.1	34	7.1	0.9	0.3	77	7.4	1.2	0.4
42	67	5.8	0.5	0.3	43	9.4	1.2	0.3	110	10.4	1.4	0.2
43	73	10.2	1.2	0.3	55	9.1	1.4	0.5	128	9.4	1.2	0.3
44	55	9.9	1.7	0.5	47	9.6	1.3	0.5	102	9.8	1.2	0.4
45	29	11.0	1.2	0.5	28	5.0	0.2	0.1	57	5.4	0.9	0.4
46	46	6.2	1.3	0.2	39	10.5	1.3	0.5	85	10.6	1.2	0.3
47	89	11.4	1.1	0.4	65	6.2	0.4	0.2	157	6.2	0.8	0.3

About 4% (n = 21) of the boys and 4.4% (n = 23) of the girls had missing teeth due to caries. Eight of the contra-lateral teeth on the left side had erupted slightly earlier that their right side counterparts (Table 2). The majority of the mandibular teeth tended to erupt earlier than the maxillary teeth with exception of second premolars and second molars (Table 2). The differences were not statistically significant (p > 0.05).

Partial correlation coefficients of tooth eruption times with height while controlling for weight were positively, but not significantly associated (p > 0.05) except for the mandibular left central incisor (# 31, p < 0.05; Table 3). On the other hand, partial correlation coefficients of tooth eruption times with weight while controlling for height were positively associated except for tooth #11, #16, #26 and #41 (Table 4). The weight of the child was significantly correlated with mean eruption times in 50% of the teeth (Table 4). When Pearson linear correlation analysis was done between tooth eruption times and BMI, there was neither a specific pattern of coefficient values nor any significant correlation between the variables.

**Table 3:** The partial correlation coefficients of tooth eruption ages of individual teeth with height while controlling for weight (n = 1041)

**Tooth type**	**Number of cases**	**Correlation coefficient**	**p-value**
11	117	0.057	0.068
12	120	0.058	0.061
13	215	0.040	0.197
14	103	0.052	0.097
15	68	0.045	0.148
16	55	0.043	0.163
17	81	0.027	0.386
21	116	0.055	0.076
22	116	0.059	0.059
23	205	0.037	0.239
24	109	0.048	0.118
25	82	0.046	0.141
26	56	0.045	0.144
27	82	0.023	0.451
31*	61	0.113	0.001
32	105	0.056	0.070
33	130	0.047	0131
34	100	0.048	0.120
35	58	0.040	0.195
36	78	0.044	0.152
37	148	0.032	0.305
41	77	0.048	0.122
42	110	0.055	0.076
43	128	0.048	0.124
44	102	0.044	0.153
45	57	0.041	0.183
46	85	0.046	0.138
47	157	0.031	0.313

**Table 4:** The partial correlation coefficients of tooth eruption ages of individual teeth with weight while controlling for height (n = 1041)

**Tooth type**	**Number of cases**	**Correlation coefficient**	**p-value**
11	117	−0.017	0.584
12	120	0.044	0.154
13	215	0.241	<0.001
14	103	0.154	<0.001
15	68	0.163	<0.001
16	55	−0.016	0.604
17	81	0.264	<0.001
21	116	0.010	0.742
22	116	0.040	0.197
23	205	0.257	<0.001
24	109	0.158	<0.001
25	82	0.162	<0.001
26	56	−0.010	0.748
27	82	0.288	<0.001
31	61	0.030	0.331
32	105	0.008	0.798
33	130	0.209	<0.001
34	100	0.220	<0.001
35	58	0.209	<0.001
36	78	0.016	0.614
37	148	0.279	<0.001
41	77	−0.030	0.338
42	110	0.014	0.658
43	128	0.202	<0.001
44	102	0.224	<0.001
45	57	0.208	<0.001
46	85	0.208	0.189
47	157	0.284	0.001

Generally, the mean eruption times of girls (Table 5) and boys (Table 6) in the present study were comparable with values from African countries (Ghana [21] and Nigeria [6]), but lower than values from countries outside Africa: Belgium [22], USA [23], Australia [24], Iran [25] and Pakistan [10].

**Table 5:** Comparison of mean eruption ages (years) of Ugandan girls with those of other nationalities in the respective year of study

**Tooth type**	**Belgium (2003)**	**Ghana (1967)**	**Nigeria (1971)**	**USA (1978)**	**Australia (2003)**	**Iran (2004)**	**Pakistan (2011)**	**Uganda (1971)**	**Uganda (Present)**
Maxillary									
Central incisor	6.9	6.0	7.1	7.2	7.2	7.6	7.5	6.2	6.2
Lateral incisor	7.9	7.3	8.0	8.2	8.2	8.8	8.4	6.9	7.2
Canine	110	9.5	10.2	11.0	11.2	12.1	107	9.3	9.3
First premolar	10.4	9.0	10.1	10.5	10.8	11.0	10.1	8.8	9.3
Second premolar	11.4	10.0	10.3	12.2	11.7	12.5	10.8	9.6	10.1
First molar	6.2	5.0	5.8	6.4	6.5	6.7	6.7	5.4	5.3
Second molar	12.0	10.9	11.4	12.1	12.3	12.5	12.0	9.8	10.7
Mandibular									
Central incisor	6.2	5.1	5.8	6.1	6.3	6.5	7.1	5.3	5.6
Lateral incisor	7.1	6.3	7.3	7.3	7.4	7.9	7.9	6.0	6.8
Canine	9.7	8.9	9.9	9.9	10.1	10.3	10.0	8.0	9.7
First premolar	10.3	9.2	9.9	10.4	10.6	11.1	10.3	8.9	9.2
Second premolar	11.4	10.3	10.6	11.1	11.7	12.6	10.8	9.8	10.2
First molar	6.2	4.4	5.8	6.3	6.3	6.7	6.5	5.4	5.2
Second molar	11.6	10.5	10.9	11.8	11.8	12.4	11.4	9.4	10.3

**Table 6:** Comparison of mean eruption ages (years) of Ugandan boys with those of other nationalities in the respective year of study

**Tooth type**	**Belgium (2003)**	**Ghana (1967)**	**Nigeria (1971)**	**USA (1978)**	**Australia (2003)**	**Iran (2004)**	**Pakistan (2011)**	**Uganda (1971)**	**Uganda (Present)**
Maxillary									
Central incisor	7.1	6.2	7.5	7.2	7.4	6.8	7.5	6.1	6.3
Lateral incisor	8.3	7.4	8.3	8.3	8.6	8.4	8.5	7.3	8.5
Canine	11.5	10.0	11.0	11.5	11.8	11.8	11.0	10.0	10.8
First premolar	10.7	9.3	10.6	11.1	11.3	12.0	10.1	9.0	9.6
Second premolar	11.6	10.3	11.1	11.7	12.1	12.0	10.1	10.5	9.5
First molar	6.3	5.0	6.3	6.5	6.7	6.8	6.7	5.1	6.4
Second molar	12.3	10.9	11.8	12.2	12.7	12.7	11.7	10.5	10.0
Mandibular									
Central incisor	6.3	5.2	6.3	6.2	6.6	6.0	6.7	5.5	6.5
Lateral incisor	7.4	6.3	7.3	7.5	7.8	7.3	8.4	6.2	5.8
Canine	10.6	9.5	10.6	10.7	11.0	9.7	11.8	9.6	10.1
First premolar	10.7	9.5	10.7	10.9	11.2	10.1	12.2	9.6	10.0
Second premolar	11.7	10.5	10.5	11.6	12.1	10.9	12.8	10.4	10.8
First molar	6.3	4.7	6.0	6.5	6.6	5.6	6.8	5.3	6.0
Second molar	11.8	10.6	11.3	12.0	12.2	11.3	12.9	10.2	11.5

The mean eruption times of girls and boys in the present study were generally higher than previously recorded in Ugandan children [13]. The biggest difference in eruption times between the present study and the previous one [13) in girls was 1.7 years in the mandibular canine (Table 5) while among the boys was 1.3 years in the mandibular second molar (Table 6). In the previous national survey [13] of the Ugandan children, tooth eruption times were generally earlier in girls with a difference of an average of 0.5 (range, 0–1.6) years. On the other hand, tooth #11, #16, #26 and #46 erupted earlier in boys.

## Discussion

In this cross sectional study, the eruption times of the permanent teeth and the anthropometric measurements were evaluated in a group of Ugandan school children aged between 4 to 15 years in Kampala. The data comprised of 1041 children with an almost equal distribution according to sex (520:521/boys: girls; Table 1). In some cultural practices indigenous to each region, data related to the date of birth might be misleading, especially if no central registration boards or no precise proof of age exist [26]. In the present study, the age of the child was ascertained by asking the child the date of birth and then confirmed with school records. The two sources of information corroborated.

The eruption or emergence of a tooth is the biological process that follows the formation of the dental crown and is essentially the penetration of the covering oral mucosa by any part of a tooth [1]. In the present study, clinical assessment of penetration of the oral mucosa by any part of the tooth was so obvious and did not require any reliability assessment of the examiners [17]. However, the reliability test of anthropometric measurement was done and gave an almost perfect agreement [18] with no evidence of systematic error observed.

The present study was school based with no radiological facilities available and consequently, we were not able to determine the level of congenitally missing teeth. Previously, Holman and Jones [27] had discussed the impact of congenitally missing teeth on the mean eruption times and concluded that estimates of eruption time without considering congenitally missing teeth were biased upward, but in any case less than 1%. Moreover, they stated that for adequate sample sizes, agenesis does not lead to substantially biased estimates. The present study recruited a moderate sample size of children (n = 1041) implying that the influence of congenitally missing teeth may not be significant.

Researchers in previous studies [1,4,28] postulated that there was a role of the endogenic, exogenic and/or environmental factors in tooth eruption time. Socioeconomic or environmental factors have been reported to directly influence nutrition with an impact on child development including tooth eruption [7,8], although Friedlaeder and Bailit [29] expressed a relative unimportance of environmental influence on eruption times of permanent teeth. Tooth eruption times in Uganda was last studied more than 40 years ago [13]. Over that period many environmental and socioeconomic changes have taken place, which included civil wars leading to internal migration and disruption of socioeconomic activities. However, with peaceful environment in the subsequent years, this was followed by an impressive economic growth [30] that improved the socioeconomic development and presumably, the nutritional status of individuals. It could be argued that these changes may partly explain the later eruption times in the present compared to the previous study [13] (Tables 5 and 6). Assessment of the role of the endogenous, exogenous or socioeconomic and environmental factors in tooth eruption was beyond the scope of the present study. Other factors that could lead to observed differences in the eruption times between the two studies are that the previous study [13] was a national estimate based on a smaller sample of children (n = 622).

Based on partial correlation analyses in the present study, the mean tooth eruption times were directly related to child height, although the relationship was generally not statistically significant (Table 3). On the other hand, eruption times were generally directly related to the weight of the child, but significant in 50% of the teeth (Table 4) indicating that the influence of weight on eruption time is non-conclusive. In a previous comparative study [31] among the Japanese children in Hiroshima, tooth eruption times were found to be directly influenced by height and weight.

In further analysis based on Pearson linear correlation, we did not observe any specific relationship between tooth eruption times and BMI probably due to conflicting outcomes of weight and height. Among the Pakistani children in Karachi, Khan [10] previously observed that tall children exhibited delayed tooth eruption irrespective of their weight while heavy and short children had early tooth eruption. He also found the eruption time of different teeth to be either directly or indirectly related to the BMI. In other studies involving first molars and incisors of Saudi male children in Riyadh and Jeddah, Khan et al. [3] observed a non significant correlation between BMI and eruption times except for tooth #32. The eruption times were generally inversely related to the BMI.

In the present study, we found a sex difference in tooth eruption times; being an average of 0.8 (range, 0–1.5) years earlier in girls (Table 2). Similarly, in the previous survey in Uganda [13], eruption times were lower in girls than boys with a difference of an average of 0.5 (range, 0–1.6) years. Additionally, we found tooth #25, #32 and #42 to have erupted earlier in boys than girls while in the previous study [13] tooth #11, #16, #26 and #46 erupted earlier in boys than girls. The reason for the differences in tooth eruption times between boys and girls is poorly understood. It is assumed that the earlier onset of the permanent dentition is part of the different sexual maturity of both sexes at a given age [32].

In the present study, about 4% and 4.4% of the boys and girls, respectively, had lost teeth due to caries. By assumption, the prevalence of extracted teeth due to caries in the deciduous dentition could have been at the same low level in these Ugandan children. Retained deciduous teeth till physiological shedding could retard the eruption of permanent teeth [13].

We found no significant differences in the mean eruption times between the teeth in the right and left side of the jaws in these Ugandan children, which corroborates findings in the previous reports [10,33].

Generally, there were no substantial differences between the mean eruption times of the children in the present study and other studies involving African populations (Tables 5 and 6). However, our results showed lower values when compared with studies from outside Africa. The major differences in eruption times among girls in both the maxillary and mandibular teeth were with the Iranian children [25] (Table 5) while among the boys, bigger differences were with the Australian [24] and Iranian children [25] in the maxillary teeth and Pakistani children [10] in the mandibular teeth (Table 6). The impact of ethnicity on the eruption process was reported in previous surveys [2,4-6] and meta-analysis [34].

## Conclusion

The mean tooth eruption times for girls were lower compared to boys except for three teeth (#25, #32 and #42). The height of the child did not show any significant influence on the tooth eruption times while the influence of weight on tooth eruption times was non-conclusive.

## Competing interests

The authors declare that they have no competing interests.

## Authors’ contributions

AK wrote the protocol, contributed to data collection and drafted the manuscript. CMR analysed the data and contributed to the manuscript writing. ER, LM and WB developed the study idea and contributed to the study design, data collection, literature review and writing of the manuscript. All the authors read and approved the final manuscript.

## Pre-publication history

The pre-publication history for this paper can be accessed here:

http://www.biomedcentral.com/1472-6831/13/15/prepub
